# Novel matrine derivative MD-1 attenuates hepatic fibrosis by inhibiting EGFR activation of hepatic stellate cells

**DOI:** 10.1007/s13238-016-0285-2

**Published:** 2016-06-24

**Authors:** Yi Feng, Hai-yan Ying, Ying Qu, Xiao-bo Cai, Ming-yi Xu, Lun-gen Lu

**Affiliations:** Department of Gastroenterology, Shanghai General Hospital, Nanjing Medical University, Shanghai, 200080 China

**Keywords:** matrine derivative, hepatic stellate cell, hepatic fibrosis, epidermal growth factor receptor, signal transduction pathway

## Abstract

Matrine (MT), the effective component of *Sophora flavescens Ait*, has been shown to have anti-inflammation, immune-suppressive, anti-tumor, and anti-hepatic fibrosis activities. However, the pharmacological effects of MT still need to be strengthened due to its relatively low efficacy and short half-life. In the present study, we report a more effective thio derivative of MT, MD-1, and its inhibitory effects on the activation of hepatic stellate cells (HSCs) in both cell culture and animal models. Cytological experiments showed that MD-1 can inhibit the proliferation of HSC-T6 cells with a half-maximal inhibitory concentration (IC_50_) of 62 μmol/L. In addition, MD-1 more strongly inhibits the migration of HSC-T6 cells compared to MT and can more effectively induce G_0_/G_1_ arrest and apoptosis. Investigating the biological mechanisms underlying anti-hepatic fibrosis in the presence of MD-1, we found that MD-1 can bind the epidermal growth factor receptor (EGFR) on the surface of HSC-T6 cells, which can further inhibit the phosphorylation of EGFR and its downstream protein kinase B (Akt), resulting in decreased expression of cyclin D1 and eventual inhibition of the activation of HSC-T6 cells. Furthermore, in rats with dimethylnitrosamine (DMN)-induced hepatic fibrosis, MD-1 slowed the development and progression of hepatic fibrosis, protecting hepatic parenchymal cells and improving hepatic functions. Therefore, MD-1 is a potential drug for anti-hepatic fibrosis.

**Electronic supplementary material:**

The online version of this article (doi:10.1007/s13238-016-0285-2) contains supplementary material, which is available to authorized users.

## **INTRODUCTION**

Hepatic fibrosis is a pathological process comprising atypical hyperplasia (dysplasia) of intrahepatic connective tissue and excessive accumulation of extracellular matrix (ECM) that can ultimately develop into hepatic cirrhosis. The main pathogenic factors that induce hepatic fibrosis include chronic hepatitis virus (i.e., HBV and HCV) and parasite infection (e.g., schistosomes), alcoholic liver disease, fatty liver disease, cholestasis, drug-induced liver injury, and autoimmune hepatitis. The persistent presence of these factors causes the production and activation of hepatic fibrosis-initiating cells, especially the activation of hepatic stellate cells (HSCs). Other cells that can be activated are fibroblasts, liver sinusoidal endothelial cells, and Kupffer cells surrounding the portal vein; mesenchymal stem cells derived from bone marrow; blood fibrocytes; and hepatocytes or biliary epithelial cells that have undergone epithelial-mesenchymal transition (EMT) (Fausther et al., 2013; Iwaisako et al., 2014b; Pinzani, 2015; Koo et al., 2016). These cells can proliferate and transdifferentiate into myofibroblasts (MFs) and then synthesize and secret a large amount of ECM, causing hepatic fibrosis and cirrhosis (Fagone et al., 2015).

Studies of the mechanisms underlying the development of hepatic fibrosis indicate that many factors associated with liver injuries can induce HSC activation (Seki and Brenner, 2015). Hepatic parenchymal cell injury can be caused by a variety of pathological factors and induce the synthesis and release of a variety of cytokines, including transforming growth factor β (TGF-β), epidermal growth factor (EGF), platelet-derived growth factor (PDGF), endothelin (ET), fibroblast growth factor (FGF), connective tissue growth factor (CTGF), and leptin, which can further activate the TGF-β/Smad, epidermal growth factor receptor (EGFR), and Ras/ERK signaling pathways to promote HSC proliferation and activity (Cuevas et al., 2011; Handy et al., 2011; Shimada et al., 2011; Liu et al., 2013). After activation, HSCs can transdifferentiate into MFs, which express α-smooth muscle actin (α-SMA), secrete ECM components (including type I and type II collagens and proteoglycan) that cause ECM accumulation, and promote the development of hepatic fibrosis. Therefore, inhibition of hepatic fibrosis through HSC inactivation is a major direction in the prevention and treatment of hepatic cirrhosis.

Two major treatments are used against hepatic fibrosis: those that eliminate pathogenic factors that cause hepatic fibrosis (e.g., antiviral therapy and glucocorticoid treatment of autoimmune or alcoholic liver diseases) and those that target ECM accumulation by decreasing its synthesis and facilitating its degradation. Clinical treatment measures for anti-hepatic fibrosis are currently limited. Therefore, it is crucial to explore new types of anti-hepatic fibrosis drugs. Matrine (MT), the effective component of *Sophora flavescens Ait*, has been shown to have anti-inflammation, immune-suppressive, anti-tumor, and anti-hepatic fibrosis activities. MT also has protective effects against acute liver injury in mice and rats (Zhang et al., 2011; Liu et al., 2014). However, the clinical pharmacological effects of this drug still need to be strengthened due to its relatively low efficacy and short half-life. In the present study, we structurally modified MT and obtained a novel thio derivative called MD-1 (C_30_N_4_H_40_SO_5_F) through thiosulfate and side chain Michael addition to prepare maleate for anti-hepatic fibrosis experiments. We examined the effects of MD-1 on HSC activation and inhibition of hepatic fibrosis in rats. In addition, the possible mechanism of action of this novel MT derivative was investigated to determine if it is a potential clinical treatment option for hepatic fibrosis.

## **RESULTS**

### **Inhibition of HSC-T6 cell proliferation by novel thio derivatives of MT**

**Figure 1:**
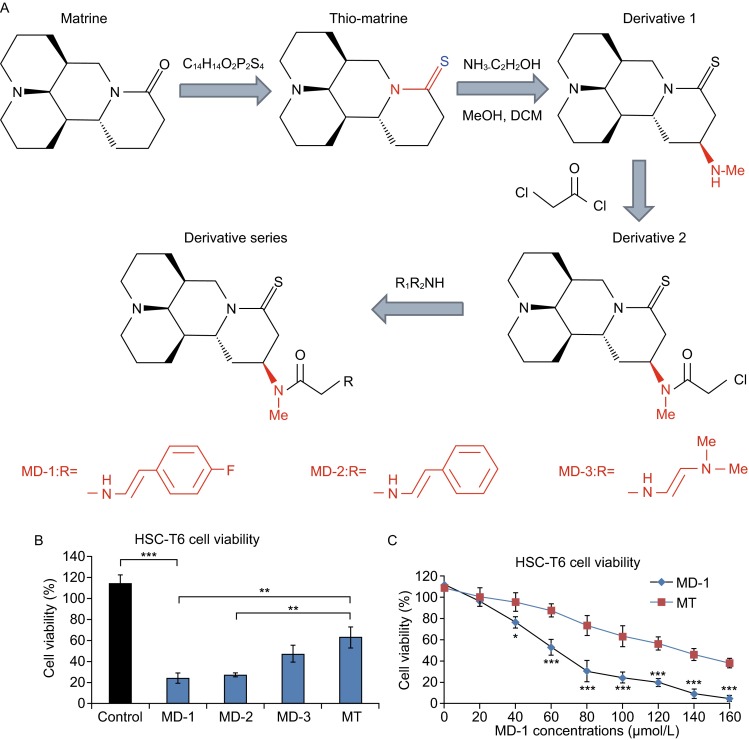
**Novel thio derivatives of MT inhibit the proliferation of HSC-T6 cells**. (A) The chemical structure of MD-1, MD-2, and MD-3. (B) The inhibitory effects of MD-1, MD-2, and MD-3 on the proliferation of HSC-T6 cells (MD-1 group: 24.2% ± 5.0%; MD-2 group: 27.4% ± 1.6%; MD-3 group: 47.2% ± 8.1%; MT group: 62.9% ± 10.0%; control group: 114.2% ± 8.5%; *n* = 3 in each group). (C) A concentration-dependent plot of the effect of MD-1 and MT on the proliferation of HSC-T6 cells. IC_50_ of MD-1 and MT was 62 µmol/L and 128 µmol/L, respectively

A series of novel thio derivatives of MT, including MD-1, MD-2, and MD-3, were obtained from structural modifications to MT (Fig. 1A, also see Supplementary Information for details). First, we determined the effects of MT and its derivatives on the proliferation of HSC-T6 cells at a concentration of 100 µmol/L. MT treatment decreased the survival rate to 62.9% ± 10.0% (*n* = 3) compared to control (114.2% ± 8.5%, *n* = 3; *P* < 0.001, Fig. 1B). Among the three derivatives of MT, MD-1 had the strongest inhibitory effect on HSC-T6 cells, decreasing the cell survival rate to 24.2% ± 5.0%, which is significantly lower than that of MT (*n* = 3; *P* = 0.004). For MD-2 the cell survival rate was 27.4% ± 1.6% (*n* = 3; *P* < 0.01 compared to MT). The inhibitory effect of MD-3 on HSC-T6 cells was equivalent to that of MT (47.2% ± 8.1%, *n* = 3; *P* = 0.10). We also examined the effects of different concentrations of MD-1 on the proliferation of HSC-T6 cells. The survival rates of HSC-T6 cells decreased as the MD-1 concentration increased. The IC_50_ values of MD-1 and MT on HSC-T6 cells were 62 µmol/L and 128 µmol/L, respectively (Fig. 1C).

### **Inhibitory effects of MD-1 on HSC-T6 cell motility**

**Figure 2:**
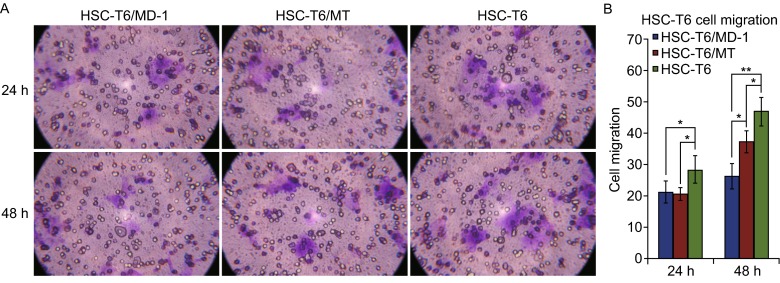
**Inhibitory effects of MD-1 on the motility of HSC-T6 cells**. (A) Top: Representative picture to show the migration ability of HSC-T6 cells at the same cell viability (50%) after 24 h of drug action (MD-1, MT, and control). Bottom: After 48 h of drug action. (B) Statistics for the migration ability of HSC-T6 cells. *P < 0.05; **P < 0.01

The inhibitory effects of MD-1 and MT on the migration of HSC-T6 cells were observed using the Transwell assay. To exclude the influence of inhibited cell proliferation, the experimental concentrations of MD-1 and MT were set at their corresponding IC_50_ values to compare their effects on cell migration at the same cell survival rate (50%). After 24 h of treatment, the difference in HSC-T6 cell migration between 62 µmol/L MD-1 and 128 µmol/L MT was not significant, but both significantly reduced motility compared to control (Fig. 2B; *P* < 0.05). However, after 48 h, the inhibitory effect of MD-1 on cell migration was significantly stronger than that of MT (*P* < 0.05, *n* = 3, Fig. 2A and 2B). Comparing the difference between 24 h and 48 h groups, the results showed that there was a significant different increase in MT-treated cells at 48 h compared with that at 24 h (*P* < 0.01), but not in MD-1-treated cells, suggesting a stronger effect of MD-1 on inhibiting HSC-T6 cell migration.

### **Effects of MD-1 on cell cycle and apoptosis in HSC-T6 cells**

**Figure 3:**
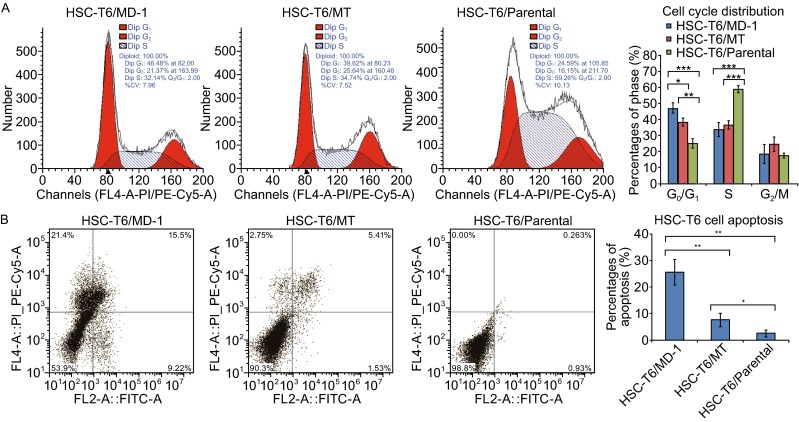
**Inhibition of cell cycle and induction of apoptosis by MD-1 in HSC-T6 cells**. (A) Left: The cell cycle distribution of HSC-T6 cells in the presence of MD-1 or MT at 62 μmol/L was examined by flow cytometry. Right: Statistics for cell cycle distribution. *P < 0.05; **P < 0.01; ***P < 0.001. (B) Left: The cell apoptosis rate was detected with flow cytometer in MD-1, MT and control group. *P < 0.05; **P < 0.01; ***P < 0.001

The effects of MD-1 on cell cycle and apoptosis in HSC-T6 cells were detected by flow cytometry. Compared to the control group, MD-1- and MT-treated HSC-T6 cells had an increased percentage of cells in G_0_/G_1_ phase and a decreased percentage of cells in the S phase (Fig. 3A). The change in the cell cycle induced by MD-1 was even more significant than the change induced by MT (G_0_/G_1_: 47.2% ± 3.2% in MD-1 group, 38.6% ± 2.5% in MT group, and 25.2% ± 3.0% in control; S: 33.6% ± 4.5% in MD-1 group, 36.6% ± 2.6% in MT group, and 58.9% ± 2.2% in control; G_2_/M: 18.5% ± 6.0% in MD-1 group, 24.9% ± 4.5% in MT group, and 17.4% ± 1.4% in control; *n* = 3 in each group). These results suggest that MD-1 could induce G_0_/G_1_ arrest in HSC-T6 cells and decrease the number of cells entering mitosis (Fig. 3A, right). The apoptosis rate in control HSC-T6 cells was 2.5% ± 1.3% (*n* = 3). After MD-1 or MT treatment for 48 h, the apoptosis rate increased to 25.6% ± 4.8% (*n* = 3; *P* = 0.001) or 7.6% ± 2.5% (*n* = 3; *P* < 0.05), respectively. The effect of MD-1 on the induction of apoptosis in HSC-T6 cells was significantly stronger than that of MT (*P* = 0.005; Fig. 3B, right).

### **Effects of MD-1 on the EGFR signaling pathway**

**Figure 4:**
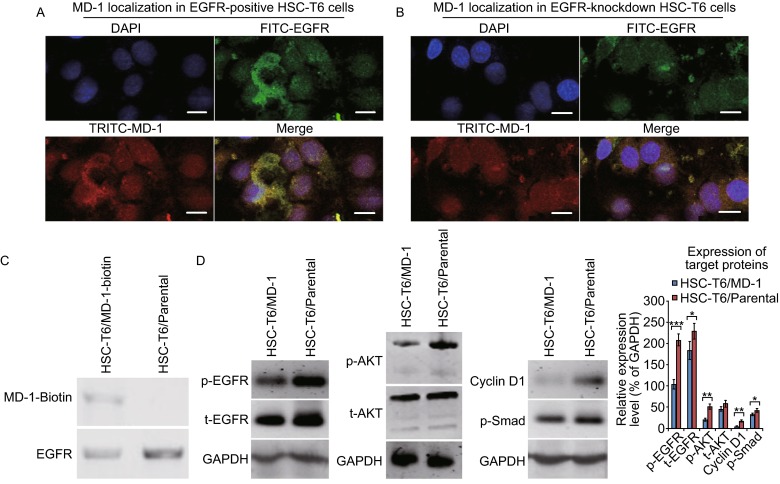
**Inhibitory effects of MD-1 on the EGFR signaling pathway in HSC-T6 cells**. (A) Immunostaining of EGFR-positive HSC-T6 cells showed MD-1 localization. Nuclei are stained with DAPI. Scale bar: 10 µm. (B) Similar to (A), but in EGFR-knockdown HSC-T6 cells. Scale bar: 10 µm. (C) Co-immunoprecipitation blots showed MD-1-Biotin was precipitated by EGFR in the MD-1-treated HSC-T6 cells, but not in the parental cells. (D) Representative Western blots to show the expression of EGFR, AKT, cyclin D1, and p-Smad. GAPDH was used as the loading control. Densitometry was performed to determine the relative expression levels normalized to GAPDH. *P < 0.05; **P < 0.01; ***P < 0.001

After biotin-labeled MD-1 was prepared, the localization of MD-1 in HSC-T6 cells and its relationship with EGFR were examined by immunofluorescence labeling and co-immunoprecipitation assay. The localization of MD-1 was consistent with EGFR on the surface of the cell membrane (Fig. 4A). After knocking down the expression of EGFR in HSC-T6 cells using an EGFR-shRNA expression plasmid, immunofluorescence labeling showed that MD-1 binding to cell membranes decreased accordingly (Fig. 4B). Co-immunoprecipitation blots also showed a positive band of MD-1-Biotin was precipitated by EGFR in the MD-1-treated cells, but not in the parental cells (Fig. 4C). These results suggest that the target molecule of MD-1 in HSC-T6 cells was EGFR and that the drug exerted its function by binding to EGFR.

We also detected the expression of EGFR downstream signaling proteins. After HSC-T6 cells were treated with MD-1, the phosphorylation levels of cell proliferation- and motility-associated EGFR and Akt significantly decreased (p-EGFR: *P* < 0.001; t-EGFR: *P* < 0.05; p-AKT: *P* < 0.01; *n* = 3 in each group, Fig. 4D), and the expression levels of cell cycle regulatory protein cyclin D1 and apoptosis-associated protein p-Smad also decreased (*P* < 0.01 and *P* < 0.05, respectively, Fig. 4D). These results suggest that the EGFR and Akt signaling pathways are key factors in regulating the molecular mechanisms by which MD-1 inhibited HSC-T6 cell proliferation, migration, and cell cycle arrest and apoptosis, and that cyclin D1 and p-Smad were also involved.

### **Effects of MD-1 on ECM synthesis and secretion in HSC-T6 cells**

**Figure 5:**
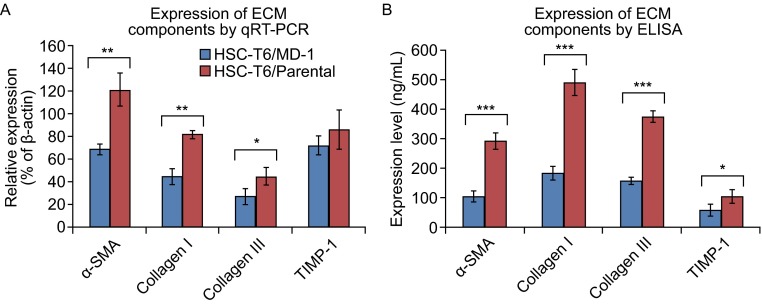
**Inhibitory effects of MD-1 on ECM synthesis and secretion in HSC-T6 cells**. (A) Relative expression levels of ECM components, including α-SMA, collagen I and III, and TIMP-1, as detected by real-time quantitative PCR (qRT-PCR). *P < 0.05; **P < 0.01. (B) Expression of ECM components in the collected cell supernatant as determined by ELISA. *P < 0.05; ***P < 0.001

After activation, HSCs transdifferentiated into MFs, which expressed α-SMA and secreted ECM. qRT-PCR demonstrated that MD-1-treated HSC-T6 cells expressed significantly decreased levels of α-SMA mRNA (*P* < 0.01, *n* = 3, Fig. 5A). The expression of intracellular type I and type III collagens was also downregulated (*P* < 0.01 and *P* < 0.05, respectively, Fig. 5A), and the expression of tissue inhibitor of metalloproteinase 1 (TIMP-1) was slightly decreased (*P* = 0.20, *n* = 3, Fig. 5A). ELISA showed that the levels of α-SMA, type I and III collagen, TIMP-1 in the MD-1-treated cells were significantly decreased (Fig. 5B). These results indicated that HSC activation could be significantly suppressed by MD-1, resulting in decreased ECM synthesis and secretion.

### **Effects of MD-1 on DMN-induced hepatic fibrosis in rats**

**Figure 6:**
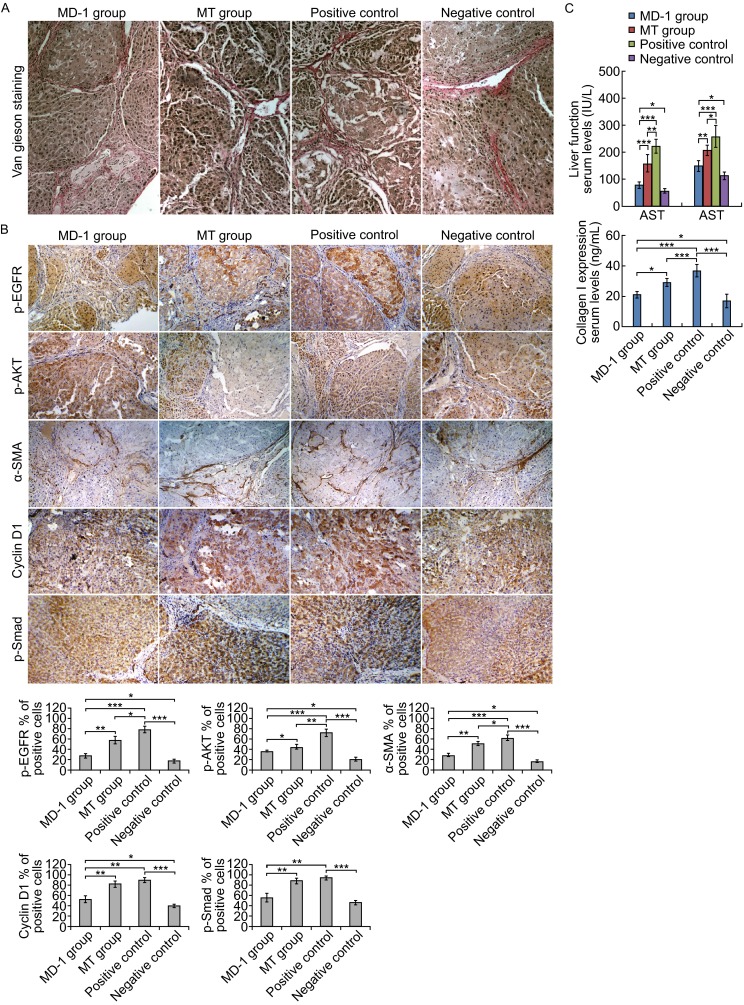
**Inhibitory effects of MD-1 on DMN-induced hepatic fibrosis in rats**. (A) The sectioned rat liver tissues were stained with Van Gieson staining reagent to show the proliferated collagen fibers (stained with red) segmented and surrounded pseudolobules in the positive control group, and MD-1 treatment significantly reduced the collagen fibers. Original magnification: 200×. (B) Top: Expression of p-EGFR, p-AKT, α-SMA, cyclin D1, and p-Smad in sectioned rat liver was examined by immunohistochemistry. The percentages of positive cells (stained with brown) were counted within five fields of view for each section using a 20× objective lens. Original magnification: 100×. Bottom: Statistics for p-EGFR, p-AKT, α-SMA, cyclin D1, and p-Smad. *P < 0.05; **P < 0.01; ***P < 0.001. (C) Top: Detection of serum ALT and AST levels on fully automated biochemical analyzer. Bottom: Detection of collagen I by ELISA. *P < 0.05; **P < 0.01; *** *P* < 0.001

A hepatic fibrosis model was established in rats by repeated intraperitoneal injection of DMN to induce hepatic fibrosis. During the initiation and progression of hepatic fibrosis, the rats were continuously treated with MD-1 or MT for 4 weeks. Two months after completing the treatment, blood and liver tissues were collected to examine liver function and hepatic fibrosis indicators. Van Gieson collagen staining of the liver tissue sections showed that DMN successfully induced hepatic fibrosis, proliferated collagen fibers segmented and surrounded pseudolobules, and hepatocytes had spotty necrosis and lymphocyte infiltration (Fig. 6A, positive control). Livers from MD-1-treated rats had significantly lower collagen fiber proliferation than controls, as well as narrow septae and incomplete segmentation of pseudolobules. However, the hepatocyte necrosis was not significant (Fig. 6A, MD-1 group). Based on pathological changes, the curative effect of MT was significantly less than that of MD-1 (Fig. 6A). Immunohistochemistry showed that the expression of p-EGFR, p-AKT, α-SMA, and cyclin D1 was significantly decreased in livers from the MD-1 group compared to the positive control group, but the expression was still higher than that of the normal control group (negative control group, Fig. 6B and 6C). Compared to the positive control group, p-EGFR, p-AKT, and α-SMA were downregulated in the MT group, whereas the reductions in cyclin D1 and p-Smad were not significant (Fig. 6B). ELISA showed that serum ALT, AST, and type I collagen levels were increased in the positive control group but significantly decreased in the MD-1 group while still being higher than the levels in the negative control group (Fig. 6C). In the MT group, ALT and AST were significantly decreased, but the reduction in type I collagen was not significant (Fig. 6C). Our results suggest a better inhibition of hepatic fibrosis in the MD-1 group than the MT group, both of which were more effective than the positive control.

## **DISCUSSION**

Hepatic cirrhosis resulting from chronic liver disease is difficult to reverse and effective clinical treatments are lacking (Tsochatzis et al., 2014; Crossan et al., 2015). Because early-stage hepatic fibrosis can be reversed, active screening of new treatment methods for early interventions for hepatic fibrosis can effectively reduce the incidence of hepatic cirrhosis, or even hepatic carcinoma, improving the quality of life for many people. Among cells involved in hepatic fibrosis, HSCs (also known as Ito cells or fat-storing cells) in the perisinusoidal space play a critical role in the initiation and development of hepatic fibrosis (Page et al., 2015; Koo et al., 2016). HSCs are activated by a variety of cytokines to proliferate and transdifferentiate into MFs and then secrete a large amount of ECM, such as collagen fibers. The interaction among cytokines, cells, and ECM promotes the initiation and progression of hepatic fibrosis, finally resulting in hepatic cirrhosis and cancer (Iwaisako et al., 2014a; Iwaisako et al., 2014b; Wallace and Friedman, 2014; Tang et al., 2015). Because the causes of the majority of liver diseases, such as chronic hepatitis B and autoimmune hepatitis, are difficult to remove, targeting the pathogenic mechanism underlying hepatic fibrosis may be an effective measure to intervene in the disease development process and treat hepatic fibrosis. If the occurrence of hepatic fibrosis can be delayed or prevented, the pathological process of hepatic injury can be attenuated or treated.

Lamivudine and entecavir are antiviral drugs used for the clinical treatment of hepatitis B that can effectively inhibit HBV replication, with stronger binding and inhibitory effects on DNA polymerase. Therefore, these drugs can inhibit the development of hepatitis and hepatic fibrosis (Papachrysos et al., 2015). Inhibition of the phosphoinositide 3-kinase (PI3K)-related signal transduction pathway using PI3K-specific inhibitor LY294002 can significantly inhibit HSC proliferation (Wang et al., 2013). Gene therapy is also an effective measure for anti-hepatic fibrosis. Treatment of a rat thioacetamide-induced hepatic fibrosis model with a human MMP-1 adenovirus plasmid was shown to significantly attenuate the degree of hepatic tissue fibrosis, decrease the hydroxyproline level and the number of active HSCs, promote hepatocyte proliferation, and improve liver tissue structure (Iimuro et al., 2003). As a novel potential method, bone marrow mesenchymal stem cell (BMSC) transplantation has become a focus of attention (Jang et al., 2015). Studies in a murine hepatic fibrosis model showed that BMSCs injected into the tail vein can reach the liver and reduce the degree of hepatic fibrosis (Sakaida et al., 2004). However, the specific mechanism underlying the differentiation of BMSCs into hepatocytes and the reduction in hepatic fibrosis is still not clear, and an influence of the local microenvironment could not be fully excluded. Because intrahepatic inflammation is persistent in the majority of patients, BMSCs circulate to the liver, where they are activated by a variety of cytokines and induced to differentiate into MFs, which become a factor in the promotion of hepatic fibrosis. Therefore, more systemic in-depth studies on gene regulation and the microenvironment in BMSC differentiation are needed to facilitate the effective control of BMSC proliferation and accurately directed differentiation, allowing BMSCs to be effectively applied in clinical treatment.

MT is a quinolizidine compound with the chemical formula is C_15_H_24_N_20_. Its molecular backbone is a heterodimeric quinolizidine ring. MT has anti-inflammatory, antiviral, anti-fibrotic, anti-arrhythmic, and immune-suppressive functions. The drug is mainly used for the treatment of viral hepatitis, hepatic fibrosis, and arrhythmia, but it also has certain efficacy in the prevention and treatment of tumors (Zhang et al., 2001; Zhang et al., 2011; Liu et al., 2014). MT can relieve pathological injury to liver parenchymal cells and non-parenchymal cells and has significant inhibitory effects on the proliferation of HSCs and fibroblasts. The extensive pharmacological activities of MT suggest that its mechanism of action is complex. A thio derivative of MT, MASM, can act directly on ribosomal protein S5 (RPS5) *in vitro* and in rats, reducing the phosphorylation levels of Ser473 and Thr308 in Akt and inhibiting HSC activation (Xu et al., 2014). However, the pharmacological activity of MT is not high, making it necessary to modify its structure to screen for derivatives that have high activities and low toxicity. The aforementioned MASM study confirmed that the pharmacological activity of a thio derivative of MT is greatly increased to that of MT and the toxicities equivalent (Hu et al., 2010; Xu et al., 2014). We performed modifications on the basis of thio derivatives of MT. The methylamino group at position 18 was acetylated to improve its stability, and an amino side chain was introduced to increase it activity. This produced the novel MT derivatives MD-1, MD-2, and MD-3, which had good stability and strong activity.

MD-1, MD-2, and MD-3 had large differences in their activities based on the different side chain groups. At 100 μmol/L, MD-1 reduced the survival rate of HSC-T6 cells to around 20%, MD-2 reduced the cell survival rate to 27.4%, and the inhibitory effect of MD-3 (47.2% cell survival rate) was equivalent to that of MT (62.9% cell survival rate, Fig. 1). These results suggest that MD-1 and MD-2 had inhibitory effects on HSC-T6 cells, whereas MD-3 did not significantly improve upon the inhibitory effect of MT on HSC-T6 cells. MD-1 significantly inhibited the proliferation and migration of HSC-T6 cells and induced G_0_/G_1_ arrest and apoptosis. Although the mechanism underlying hepatic fibrosis is complex, the multi-functional transmembrane glycoprotein EGFR specifically interacts with EGF and TGF-β1, causing its dimerization and regulating cell growth, proliferation, and differentiation (Voon et al., 2013). The EGFR-related signal transduction pathways are activated in HSCs in liver injury and chronic liver disease to promote the development and progression of hepatic fibrosis. Therefore, we focused on studying the effect of MD-1 on the EGFR-related signal transduction pathways. Immunofluorescence showed that the target molecule of MD-1 in HSC-T6 cells was EGFR. MD-1 interacted with EGFR on the surface of cell membranes, inhibiting EGFR phosphorylation. Inhibition of the phosphorylation of downstream protein kinases, such as Akt, affected the expression and activity of target proteins that regulate cell proliferation, migration, cell cycle, and apoptosis, such as cyclin D1 and p-Smad, finally changing the biological behaviors of cells. MD-1 reduced the synthesis and secretion of ECM components, such as type I collagen and type III collagen, in HSC-T6 cells, thereby exerting its anti-hepatic fibrosis activity. In the DMN-induced hepatic fibrosis model, MD-1 treatment delayed the development and progression of hepatic fibrosis, protected liver parenchymal cells, and improved liver function. Although the present study focused on the effect of MD-1 by inhibiting EGFR activation, other signaling pathways, such as the Ras/ERK pathway, may also be involved in hepatic fibrosis. Therefore, there are further studies needed to be carried out on the mechanisms of MT derivatives.

In summary, the present study reports a novel synthesized MT derivative, MD-1, that can significantly inhibit HSC activity, induce HSC apoptosis, and decrease the secretion of ECM components by HSCs. The drug has a protective effect on liver parenchymal cells in a rat DMN-induced hepatic fibrosis model. The possible mechanism by which MD-1 exerts its biological functions may be via EGFR binding on the cell surface, inhibiting its function and blocking the EGFR-related downstream signaling pathways. Thus, MD-1 is a potential clinical drug for anti-hepatic fibrosis.

## **MATERIALS AND METHODS**

### **Cell culture**

The rat HSC-T6 cell line was a gift from the Molecular Cancer Research Laboratory in the Eastern Hepatobiliary Surgery Hospital of Second Military Medical University (Xu et al., 2015). Cells were cultured in DMEM (GIBCO, New York, USA) containing 10% fetal bovine serum (FBS) in a 5% CO_2_ atmosphere at 37°C. MT and its derivatives were synthesized by the School of Pharmacy, Second Military Medical University. The powder form of each compound (2 mg) was added to 200 µL DMSO until completely dissolved, then 1800 µL ddH_2_O added to obtain a working solution of 1 mg/mL for future use.

### **Cell proliferation**

HSC-T6 cells were cultured to the logarithmic phase and then inoculated onto 96-well plates (10^4^ cells/well) for 24 h. Different gradient concentrations of MT and its derivatives MD-1, MD-2, and MD-3 were added. Each concentration group had eight replicate wells. After cells were cultured for another 24 h, cell proliferation was detected using the Cell Counting Kit-8 (CCK-8) reagent kit (Dojindo Molecular Technologies, Inc., Shanghai, China). Based on the IC_50_ values of the three drugs, MD-1, the one with the strongest activity, was selected for other experiments.

### **Cell motility**

Transwells were placed in 24-well plates and HSC-T6 cells placed in the top chamber (2 × 10^5^ cells/200 µL). The bottom chamber contained 500 µL culture medium containing 10% FBS. Cells in the top chambers were treated with MD1 or MT at the corresponding IC_50_ (62 µmol/L or 128 µmol/L, respectively). A control group without drug was used for comparison. After 48 h of culture, cells in the top layer of the Transwell were wiped and stained with 0.1% crystal violet for 15 min. Three fields were randomly selected under a light microscope (200× magnifications) for cell counting and photography. Experiments were performed in three biologically independent replicates.

### **Detection of cell cycle and cell apoptosis**

HSC-T6 cells were inoculated onto a 6-well plate at 5 × 10^5^ cells/well. MD-1 or MT was added at 62 μmol/L. After 48 h, the cells were collected and washed twice with pre-cooled PBS. Some cells were fixed in pre-cooled 75% ethanol in a 4°C refrigerator overnight, washed with PBS twice, and stained with propidium iodide (PI) containing RNase in the dark for 30 min. Cell cycle phase was detected using a flow cytometer (FACS420, BD Biosciences, San Jose, CA). The other cells were used for annexin V/PI staining. The apoptosis rate was also determined by flow cytometry.

### **Binding and inhibiting EGFR**

The shRNA plasmid pGenensil-shEGFR, targeting the rat EGFR gene, and a negative control vector, pGenesil-shMC, were constructed by Wuhan Genesil Biotechnology Co., Ltd. (Wuhan, China). The 21-nt sequence of EGFR-shRNA targeted base pairs 2268–2290 (5′-gga tat taa agg aaa cag aat-3′) of the EGFR gene (GenBank: M37394.2). A mock control shRNA vector (shMC: 5′-gac ttc ata agg cgc atg cat-3′) was concomitantly constructed. HSC-T6 cells were inoculated on a 6-well plate at 5 × 10^5^ cells/well. The shRNA plasmids were transfected into HSC-T6 cells using Lipofectamine 2000 (Invitrogen Corporation Shanghai Representative Office, Shanghai, China). After 48 h of continuous culture, cells were screened using G418 (400 μg/mL). The obtained cells were named HSC-T6-shEGFR and HSC-T6-shMC cells.

HSC-T6, HSC-T6-shEGFR, and HSC-T6-shMC cells were inoculated onto 6-well plates at 5 × 10^5^ cells/well. Biotin-labeled MD-1 (MD-1-Biotin) was added at a concentration of 62 μmol/L. After culturing for another 48 h, the cells were collected. A part of cells were lysed in RIPA protein lysis buffer and the harvested proteins subjected to Western blot for detection of EGFR expression. A part of cells were smeared onto slides for immunofluorescence double labeling to localize MD-1-Biotin and EGFR. The working concentration of FITC-conjugated goat anti-rat EGFR and TRITC-conjugated anti-biotin antibodies was 1:1000 (Cell Signaling Technology, Inc., Beverly, MA). The rest of cells were prepared for the co-immunoprecipitation assay using the corresponding specific antibodies for MD-1-Biotin and EGFR.

### **Detection of EGFR signal transduction pathway-associated proteins**

HSC-T6 cells were inoculated onto 6-well plates at 5 × 10^5^ cells/well. MD-1 was added at a concentration of 62 μmol/L. After 48 h, the cells were collected and lysed in RIPA protein lysis buffer (Life Technologies Corporation, New York, USA). Total protein was extracted for Western blot detection of downstream proteins in the EGFR signal transduction pathway. The primary antibodies included EGFR, p-EGFR, AKT, p-AKT (Cell Signaling Technology, Danvers, MA), Survivin, cyclin D1 (Abcam Inc., Cambridge, MA), and p-Smad (Santa Cruz Biotech Inc., CA).

### **Detection of ECM proteins**

HSC-T6 cells were inoculated onto 6-well plates at 5 × 10^5^ cells/well. MD-1 was added at a concentration of 62 μmol/L. After 48 h, the cells and culture supernatant were collected. Cells were treated with TRIzol (Life Technologies Corporation, New York, USA) to extract total RNA for real-time quantitative RT-PCR (qRT-PCR) of ECM indicators using the PrimeScript RT Reagent Kit (TaKaRa Inc., Dalian, China). The primer sequences were: β-actin (537 bp), upstream 5′-ACC CAC ACT GTG CCC ATC TAT G-3′ and downstream 5′-AGA GTA CTT GCG CTC AGG A-3′; α-SMA (120 bp), upstream 5′-CCG AGA TCT CAC CGA CTA CC-3′ and downstream 5′-TCC AGA GCG ACA TAG CAC AG-3′; type III collagen (438 bp), upstream 5′-AGG CCA ATG GCA ATG TAA AG-3′ and downstream 5′-TAT TGG GTG AA A CAG CA-3′; TIMP-1 (250 bp), 5′-TCC CCA GAA ATC GAG AC-3′ and downstream 5′-TCA GAT TAT GCC AGG GAA CC-3′. The cell culture supernatant was used to detect ECM indicators by ELISA (Shanghai Westang Bio-Tech Co., Ltd, Shanghai, China).

### **Construction of the rat hepatic fibrosis model**

A total of 20 male SD rats (Shanghai SLAC Laboratory Animal Co., Ltd, Chinese Academy of Sciences, Shanghai, China) at 4 weeks of age and a body weight of 80–100 g were randomly divided into four groups of five animals each: normal control group, model control group, MT group, and MD-1 group. Except for the normal control group, the rats were intraperitoneally injected with 1% DMN solution (10 μg/kg). Injections were performed three times each week (Monday, Wednesday, and Friday) for four consecutive weeks. The normal control group received an equal volume of normal saline. Starting from week 5, rats in the MT and MD-1 groups were treated with the drug of interest via intragastric administration (62 μmol/L/kg) three times each week (Monday, Wednesday, and Friday) for four consecutive weeks. The blank control group and model control group received of an equal volume of normal saline via intragastric administration. After the drug treatment was finished, animals were continuously fed for 2 months.

Rats were sacrificed after anesthesia. Blood and liver tissues were collected for evaluation of liver function and liver fibrosis indicators. The liver was fixed in 10% formalin for 6 h, embedded in paraffin, and sectioned. Staining was performed using the Van Gieson staining reagent kit (Maxim Biotechnology Development Co. Ltd, Fuzhou, China) according to the manufacturer’s instructions. Fibrosis was observed under a microscope. p-EGFR, p-AKT, α-SMA, cyclin D1, and p-Smad were detected by immunohistochemistry. Serum ALT, AST, and type I collagen were detected by ELISA. All animal experiments were approved by the Animal Ethics Committee of Second Military Medical University (Shanghai, China).

### **Statistical analysis**

Experimental data are presented as mean ± SD. One-way ANOVA was performed using SPSS (version 18.0). The least significant differences (LSD) test was performed if variances were homogeneous, and Dunnett’s T3 method was used if variances were heterogeneous. A *P* value < 0.05 was considered significant.

## Electronic supplementary material

Below is the link to the electronic supplementary material.

Supplementary material 1 (PDF 560 kb)
