# Supplementary material for: Novel matrine derivative MD-1 attenuates hepatic fibrosis by inhibiting EGFR activation of hepatic stellate cells
Source: Protein Cell. 2016 Jun 24;7(9):662–72. doi: 10.1007/s13238-016-0285-2 (PMC5003784; doi:10.1007/s13238-016-0285-2)
Supplement: Supplementary file 1 — Supplementary material 1 (PDF 560 kb) [file 13238_2016_285_MOESM1_ESM.pdf]

Supplementary Information

I. The synthesis of matrine derivative

1. Roadmap

Matrine is a kind of alkaloid from quinolizidine which composed of four hexatomic rings. The anti-fibrotic activity of matrine is closely related to its chemical structure. The 1-amino is of critical importance to maintain the activity of the main ring. The 15- and 16-amidations are required for its activity. Replacing the 15-oxygen with thio can significantly up-regulate matrine's activity and introduction of side chains at 13- and 14-position could also help to enhance its activity (Figure S1).

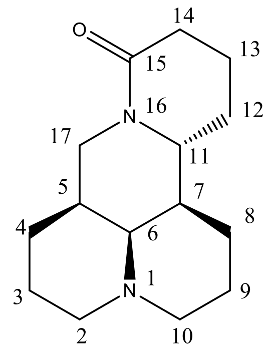

Figure S1 Chemical structure of matrine

Structural modifications of matrine (with Sophocarpine as core nucleus) by Lawson reagent ($C_{14}H_{14}O_2P_2S_4$) can get the intermediate (Thio-Matrine). By Michael addition in a solution of ammonia in ethanol, thio-matrine can further transmute into Derivative 1. Derivative 2 can be obtained from Derivative 1 by aminoacylation of chloroacetyl chloride. Finally, by substitution reactions with a series of substituted ammonia, a series of matrine derivatives, such as MD-1, MD-2, and MD-3，can be synthesized (Figure S2).

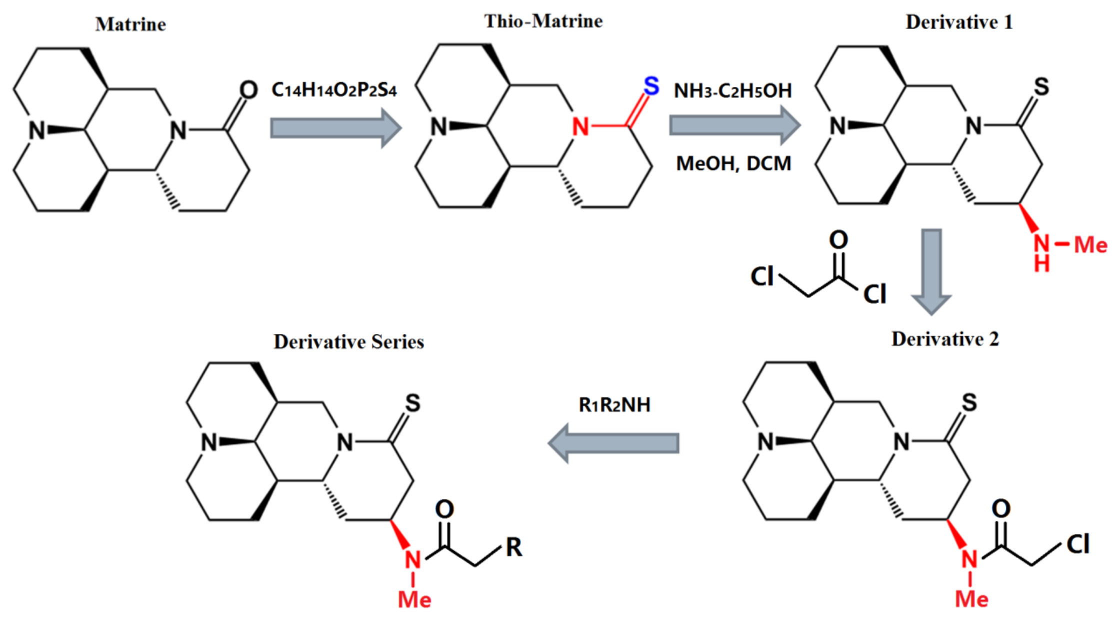

Figure S2. Synthesis of matrine derivatives

2. NMR and ESI-MS spectral data of matrine derivatives

Derivatives	^{1}H、^{13}C NMR(TMS,ppm)、MS（ESI，m/z）
MD-1	^{1}H NMR (600 MHz, $CDCl_3$) δ 7.40 – 7.34 (m, 2H), 7.07 (t, J = 8.7 Hz, 2H), 5.44 (dd, J = 11.9, 3.8 Hz, 1H), 4.93 (s, 1H), 4.46 (s, 1H), 3.92 – 3.79 (m, 2H), 3.58 (t, J = 12.2 Hz, 1H), 3.52 – 3.39 (m, 2H), 3.31 (dd, J = 18.2, 4.4 Hz, 1H), 3.16 – 3.00 (m, 1H), 2.98 – 2.78 (m, 5H), 2.28 (d, J = 23.1 Hz, 1H), 2.14 – 1.90 (m, 8H), 1.78 (d, J = 11.9 Hz, 2H), 1.67 – 1.44 (m, 5H).<br>^{13}C NMR (151 MHz, $CDCl_3$) δ 194.48, 170.64, 160.73, 135.00, 129.30, 129.25, 114.80, 114.66, 76.76, 76.55, 76.34, 63.28, 56.80, 56.46, 52.42, 51.04, 49.82, 43.45, 42.72, 40.94, 35.42, 27.78, 27.06, 26.58, 26.11, 20.65, 20.02.<br>MS (ESI): m/z 459.59$[M+H]^+$
MD-2	^{1}H NMR (600 MHz, $CDCl_3$) δ 7.36 – 7.33 (m, 2H), 7.26 (dd, J = 15.8, 7.4 Hz, 3H), 5.43 (dd, J = 11.9, 4.0 Hz, 1H), 4.81 (dd, J = 132.2, 121.5 Hz, 1H), 4.24 (d, J = 225.0 Hz, 1H), 3.57 (t, J = 12.1 Hz, 1H), 3.50 – 3.43 (m, 2H), 3.29 (dd, J = 18.0, 4.1 Hz, 1H), 3.14 – 2.99 (m, 1H), 2.98 – 2.90 (m, 2H), 2.87 (dd, J = 22.7, 17.6 Hz, 7H), 2.28 (s, 1H), 2.13 – 2.03 (m, 3H), 2.03 – 1.85 (m, 5H), 1.83 – 1.71 (m, 2H), 1.68 – 1.44 (m, 5H).<br>^{13}C NMR (151 MHz, $CDCl_3$) δ 194.50, 170.65, 139.46, 128.19, 127.97, 125.68, 63.25, 56.81, 56.46, 51.04, 50.82, 43.44, 42.63, 40.93, 36.22, 35.44, 27.77, 27.08, 26.59, 26.12, 20.67, 20.03.<br>MS (ESI): m/z 455.24$[M+H]^+$
MD-3	^{1}H NMR (600 MHz, $CDCl_3$) δ 5.43 (dd, J = 11.9, 4.0 Hz, 1H), 4.71 (d, J = 242.8 Hz, 1H), 4.48 – 4.06 (m, 1H), 3.57 (t, J = 12.3 Hz, 1H), 3.54 – 3.44 (m, 2H), 3.30 (dd, J = 17.8, 4.3 Hz, 1H), 3.11 – 2.98 (m, 1H), 2.94 – 2.83 (m, 5H), 2.83 – 2.72 (m, 2H), 2.60 – 2.45 (m, 4H), 2.33 (s, 5H), 2.28 (d, J = 5.0 Hz, 1H), 2.19 – 1.86 (m, 7H), 1.83 – 1.71 (m, 2H), 1.67 – 1.45 (m, 5H).

	^{13}C NMR (151 MHz, $CDCl_3$) δ 194.52, 170.69, 63.25, 58.63, 56.82, 56.45, 51.04, 50.65, 46.65, 44.93, 43.45, 42.67, 40.91, 35.43, 27.82, 27.07, 26.59, 26.11, 20.65, 20.01.
